# Detrusor underactivity is associated with metabolic syndrome in aged primates

**DOI:** 10.1038/s41598-023-33112-3

**Published:** 2023-04-25

**Authors:** Natalia P. Biscola, Petra M. Bartmeyer, Kari L. Christe, Ricki J. Colman, Leif A. Havton

**Affiliations:** 1 Department of Neurology, Icahn School of Medicine at Mount Sinai, New York, NY USA; 2 California National Primate Research Center, University of California at Davis, Davis, CA USA; 3 Wisconsin National Primate Research Center, University of Wisconsin-Madison, Madison, WI USA; 4 Department of Cell and Regenerative Biology, University of Wisconsin-Madison, Madison, WI USA; 5 Departments of Neurology and Neuroscience, Icahn School of Medicine at Mount Sinai, 1468 Madison Avenue, New York, NY 10029 USA; 6 James J. Peters Veterans Affairs Medical Center, Bronx, NY USA

**Keywords:** Metabolic syndrome, Bladder

## Abstract

Lower urinary tract (LUT) dysfunction is prevalent in the elderly population, and clinical manifestations include urinary retention, incontinence, and recurrent urinary tract infections. Age-associated LUT dysfunction is responsible for significant morbidity, compromised quality of life, and rising healthcare costs in older adults, but its pathophysiology is not well understood. We aimed to investigate the effects of aging on LUT function by urodynamic studies and metabolic markers in non-human primates. Adult (n = 27) and aged (n = 20) female rhesus macaques were evaluated by urodynamic and metabolic studies. Cystometry showed detrusor underactivity (DU) with increased bladder capacity and compliance in aged subjects. Metabolic syndrome indicators were present in the aged subjects, including increased weight, triglycerides, lactate dehydrogenase (LDH), alanine aminotransferase (ALT), and high sensitivity C-reactive protein (hsCRP), whereas aspartate aminotransferase (AST) was unaffected and the AST/ALT ratio reduced. Principal component analysis and paired correlations showed a strong association between DU and metabolic syndrome markers in aged primates with DU but not in aged primates without DU. The findings were unaffected by prior pregnancies, parity, and menopause. Our findings provide insights into possible mechanisms for age-associated DU and may guide new strategies to prevent and treat LUT dysfunction in older adults.

## Introduction

Age-associated lower urinary tract (LUT) impairments commonly result in an incomplete bladder emptying, progressive urinary retention, incontinence, need for bladder catheterization, and a markedly increased risk for urinary tract infections requiring outpatient antibiotic treatment or hospitalization^1,2^. Both over- and underactive bladder syndromes are prevalent with aging, contribute to impaired bladder emptying, and are responsible for significant morbidity, compromised quality of life, and rising healthcare costs in a rapidly growing older adult population^3–6^. LUT dysfunction therefore represents an important threat to the medical and financial well-being of older adults, affected families, and society, as care for incontinence and urinary retention are critical risk factors for the loss of independent living and need for long-term care in skilled nursing facilities and similar forms of assisted living^7^.

Several medical conditions are associated with the development of LUT dysfunction in the aging population and includes stroke, Parkinson’s disease, multiple sclerosis, diabetes mellitus, and bladder outlet obstructive syndrome, in addition to normative aging^8,9^. Both overactive and underactive forms of bladder dysfunction may develop with aging, either separately or in a mixed clinical presentation, but our knowledge about the underlying pathologic processes for the different types of detrusor impairments and how they may be inter-related is sparse^10–12^. Recent national and international expert panels have therefore recommended expanded research into the mechanisms of spontaneous detrusor dysfunction associated with aging and neurological impairments as well as into a better understanding of linkages between LUT dysfunction in older adults and other geriatric syndromes^2,13,14^. For this purpose, identification of clinically relevant animal models, which are suitable for translational research, will be of critical importance for moving basic discoveries to the clinic.

Rhesus macaques (*Macaca mulatta*) share about 95% of genetic homology and a close phylogenetic relationship with humans^15,16^. Rhesus monkeys and humans also show a similar physiology for multiple organs and body functions, including the immune, musculoskeletal, and reproductive systems^16^. The menstrual cycle length and post-menopausal hormonal changes are very similar in rhesus macaques and humans^17,18^. Both humans and rhesus macaques are also at risk for similar age-related medical conditions, such as obesity, metabolic syndrome, and diabetes mellitus^16,19–21^. Rhesus macaques are therefore well positioned for translational research studies on aging and age-related medical complications. However, studies of LUT function in aging non-human primates (NHPs), including rhesus macaques, have been sparse or largely absent.

## Methods

All animal care and data collection, including urodynamic recordings, for these research studies were performed at the California National Primate Research Center (CNPRC), University of California at Davis, and at the Wisconsin National Primate Research Center (WNPRC), University of Wisconsin-Madison. Both academic institutions are accredited by the Association for Assessment and Accreditation of Laboratory Animal Care (AAALAC) International. All animal research study protocols were approved by the Institutional Animal Care and Use Committee (IACUC) at UC Davis and UW Madison, and all animal procedures and care was carried out in compliance with the *Guide for the Care and Use of Laboratory Animals* provided by the Institute for Laboratory Animal Research (2011)^22^. Guidelines for ARRIVE 2.0 for the care and use of laboratory animals were also followed^23^.

### Animals

A total of 47 female rhesus macaques *(Macaca mulatta)* were included in the studies. The subjects were divided into adult and aged groups, and a subject was considered aged when 20 years old or older based on established criteria for rhesus macaques^16^. CNPRC contributed with all adult animals (n = 27) and 14 aged rhesus macaques, whereas WNPRC contributed with 6 aged animals. The adult animals were 3.9–14.9 years old (n = 27) and aged subjects were 20.0–31.8 years of age (n = 20). All adult females were within normal reproductive age, and showed an active menstrual cycle with cyclical genital bleeding. For the aged cohort, 6 of 20 females were post-menopausal, a functional state determined by documented amenorrhea for over 12 months. Both nulliparous females and animals with prior pregnancy and birth history were included in both groups. Body weight was obtained from all animals on day of urodynamic studies. Body fitness and condition was determined using a body condition scoring (BCS) system, which incorporates both obesity and muscularity on a scale from 1 to 5^24–26^.

### Urodynamic recordings

The urodynamic studies were performed according to our established procedures for cystometrogram (CMG) recordings in rhesus macaques^27,28^. All subjects were first sedated by an intramuscular (IM) injection of ketamine (10 mg/kg) followed by the placement of an intravenous (IV) catheter for the administration of anesthetics and fluid. An endotracheal tube was placed for airway protection. Ketamine administration was next provided by constant rate infusion (CRI) at 10–12 mg/kg/hour IV with adjustments of the infusion rate to maintain sedation and immobilization at the lightest plane of ketamine anesthesia possible.

For urodynamic studies, a triple-lumen 7-Fr transurethral bladder catheter or double-lumen 6-Fr transurethral catheter was placed (Laborie Medical Technologies, Corp, Williston, VT). The cystometry and urethral pressure profile catheter ports were individually attached to a TSD 104A pressure transducer system and connected to an MP 150 Data Acquisition System (Biopac Systems, Goleta, CA). For cystometrogram (CMG) recordings, the bladder was initially emptied and subsequently partially filled with saline at a target rate of 50–60 ml/min, using the fill port of the triple lumen catheter. The bladder pressure was continually monitored and increased from a baseline pressure of 0–5 cm H_2_O to a target bladder pressure of 20–25 cm H_2_O. The bladder filling was stopped when the target net increase of approximately 20 cm H_2_O was achieved. No voiding was seen at the end of the bladder filling procedure. Instead, a delayed reflex detrusor contraction was typically evoked by the partial bladder filling, resulting in a further increase of bladder pressure, and voiding was usually observed within a delay of 30–60 s after the completion of the partial bladder filling. CMG recordings documented the bladder and urethral pressure changes associated with bladder filling, onset of the micturition reflex, and voiding. After completion of the CMG recordings, all animals recovered well from the procedure and returned to normal baseline activities.

### Urodynamic analysis

A comprehensive set of quantitative outcome measures was collected from the CMG recordings. The baseline bladder pressure after bladder emptying (Pbase), bladder pressure at the completion of partial bladder filling with saline (Pfill), and change in bladder pressure from partial bladder filling (P∆fill) were first determined. In addition, the duration of partial bladder filling by saline infusion was measured, and the infusion rate of partial bladder filling with saline was calculated. The infused volume of saline to increase bladder pressure about 25 cmH_2_O above Pbase (IV25) was determined, and bladder compliance was calculated as IV25/P∆fill (Bcomp). Time between end of bladder infusion and onset of reflex voiding (Vdelay) was calculated. Bladder pressure at onset of voiding (Pvoid), and peak pressure during detrusor contraction (Ppeak) were measured, and the increase in pressure from end of bladder filling to peak pressure during detrusor contraction was calculated as Ppeak − Pfill. The duration of voiding (Vdur) and voided volume (Vv) were measured, and flow rate of voiding was calculated as Vv/Vdur. Voiding efficiency was calculated as Vv/infused bladder volume (VE), and post-void residual volume was determined from the withdrawal volume of bladder contents after voiding (PVR). Capacity-IV25 was calculated as the sum of the voided volume and PVR following partial filling of an emptied bladder to a volume that resulted in a 20–25 cmH_2_O raise in bladder pressure.

### Clinical biochemistry panels

A comprehensive clinical biochemistry panel was obtained as part of study entry evaluation for adult and aged subjects. All animals were fasted for a minimum of 16 h before the blood draw for sample collection. The clinical biochemistry panel included serum sodium (Na), potassium (K), chloride (Cl), total CO_2_, anion gap, phosphorous, calcium, BUN, creatinine, glucose, total protein, albumin, alanine aminotransferase (ALT), aspartate aminotransferase (AST), creatine phosphokinase (CPK), alkaline phosphatase (Alk Phos), gamma-glutamyl transferase (GGT), lactate dehydrogenase (LDH), cholesterol, triglyceride, total bilirubin, direct bilirubin, and high-sensitivity C-reactive protein (hsCRP). There were some differences in the composition of a comprehensive metabolic panel between the CNPRC and WNPRC sites to account for some differences in the number of observations for laboratory outcome measures, but all available data were included in the analyses.

### Statistical analysis

All data are presented as mean ± standard error (SE). GraphPad Prism, version 7.05, (GraphPad Software, Inc, La Jolla, CA), was used, initially to test data distribution for normality and subsequently for t-test and non-parametric Mann Whitney U-Test studies to compare data sets between groups. A value of *p* < 0.05 was considered to reflect a statistically significant difference between groups. For comparing two proportions, the N-1 Chi-squared test was performed using the MedCalc Comparison of Proportions Calculator (MedCalc Software, Ostend, Belgium).

Principal component analysis (PCA) was performed to compress a group of related variables into a new single entity, component, using the Matlab function “pca”. Any missing values in the dataset were treated using the Matlab function “missingvalues”, and the function “zscore” was applied to avoid scaling problems. The code used to perform these tests is available in Github (https://github.com/petrabartmeyer/PCA_analysis).

A correlation matrix analysis provided a pairwise study between the variables for each subgroup. The correlation matrix was calculated using the Matlab function “corrcoef”. The values in the correlation matrix were in the interval [− 1, 1], where values below zero represent a negative correlation between variables and values above zero represent positive correlation between variables. As values approach − 1 or 1, the correlation strength is increased. The code used to perform the correlation matrix analysis is available in Github (https://github.com/petrabartmeyer/PCA_analysis).

## Results

### Urodynamic studies in adult and aged female rhesus macaques

**Figure 1:**
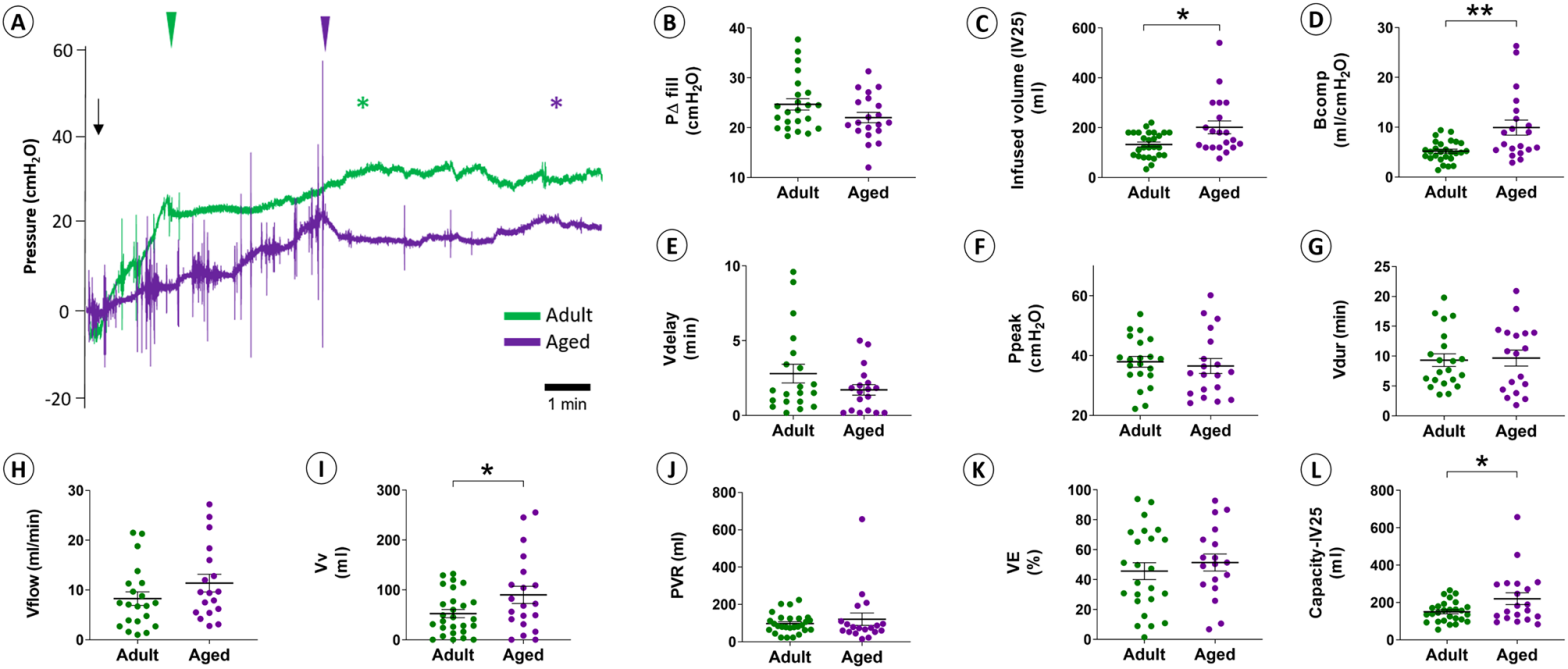
Urodynamic studies in adult and aged rhesus macaques. Reflex micturition was evoked in female subjects and shown by filling cystometry and pressure flow studies (**A**). Following emptying of the bladder, saline was infused to raise bladder pressure about 25 cm H_2_O above baseline (P∆ fill). The black arrow indicates start of bladder infusion, and the green and purple arrow heads indicate end of infusion in representative adult and aged subjects, respectively. Green and purple asterisks show onset of voiding in each of the two subjects. Transient pressure spikes reflect movement artifacts during bladder infusion. Note similar pattern of delayed onset of evoked voiding after partial bladder filling to similar pressures in both subjects. A total of 110 ml was infused in the adult subject, and 240 ml was infused in the aged animal to reach the target bladder pressure elevation. Statistical analysis showed significant elevation of IV25 (*p* < 0.05), Bcomp (*p* < 0.01), Vv (*p* < 0.01), and Capacity-IV25 (*p* < 0.01) (**C**, **D**, **I**, **L**) in the aged cohort compared to adult animals, whereas there was no detectable difference in P∆ fill, Vdelay, Ppeak, Vdur, Vflow, PVR, or VE between the groups (**B**, **E**, **F**, **G**, **H**, **J**, **K**). Data are presented as mean ± SE, and statistics are shown as * indicating *p* < 0.05 and ** indicating *p* < 0.01 between groups.

Comprehensive urodynamic studies of passive detrusor properties and evoked micturition reflexes were performed in 27 adult female and 20 aged female rhesus macaques. The studies included filling cystometry and pressure flow studies (Fig. 1).

#### Filling cystometry

An emptied bladder was partially filled with saline to a target pressure of 20–25 cmH_2_O above baseline pressure (P∆ fill) using the fill port of a multi-lumen transurethral catheter in adult and aged subjects (Fig. 1A). P∆ fill was 24.7 ± 1.1 cmH_2_O in the adults (n = 23) and 22.0 ± 1.0 cmH_2_O in the aged subjects (n = 20) (Fig. 1B). The increase in bladder pressure was not statistically different between the two groups. The infused volume (IV25) to achieve P∆ fill was 201.1 ± 25.6 ml in the aged group (n = 20) and significantly larger than the corresponding volume for the adult subjects of 132.5 ± 9.7 ml (n = 27, *p* < 0.05) (Fig. 1C). Bladder compliance (Bcomp) was also significantly higher at 9.9 ± 1.5 ml/cm H_2_O in the aged group (n = 20) compared to a Bcomp of 5.2 ± 0.4 ml/cm H_2_O in the adult group (n = 27, *p* < 0.01) (Fig. 1D).

#### Pressure flow studies

The micturition reflex was evoked by the partial bladder filling with subsequent detrusor contractions and start of voiding (Vdelay) at 2.8 ± 0.6 min and 1.7 ± 0.4 min after the end of the infusion in adult (n = 20) and aged (n = 18) subjects, respectively (Fig. 1E). There was no statistical difference between the groups. The peak bladder pressure (Ppeak) was 37.9 ± 1.8 cm H_2_O in the adult group (n = 21) and not statistically different from the corresponding Ppeak at 36.5 ± 2.5 cm H_2_O in the aged group (n = 19) (Fig. 1F). The voiding duration (Vdur) was 9.3 ± 1.0 min for the adult group (n = 21) and not statistically different from the corresponding Vdur of 9.7 ± 1.3 min in the aged group (n = 18) (Fig. 1G). The voiding flow rate (Vflow) was 8.3 ± 1.4 ml/min for the adult group (n = 21) and not statistically different from the corresponding Vflow of 11.4 ± 1.8 ml/min in the aged group (n = 18) (Fig. 1H). The voided volume (Vv) was 89.8 ± 17.2 ml in aged subjects (n = 20) and significantly larger than the Vv of 52.3 ± 8.3 ml in adults (n = 27, *p* < 0.05) (Fig. 1I). The post-void residual volume (PVR) was 97.6 ± 10.3 ml for the adult group (n = 27) and not statistically different from the corresponding PVR of 121.2 ± 33.0 ml for the aged group (n = 19) (Fig. 1J). The voiding efficiency (VE) was 45.6 ± 5.7 ml in adults (n = 24) and not significantly different from the VE in the aged subjects at 51.4 ± 5.7 (n = 18) (Fig. 1K). Capacity-IV25 was determined as the added sum of Vv and the post-void residual volume (PVR) and was significantly larger at 220.0 ± 31.8 ml in the aged subjects (n = 20) compared to the Capacity-IV25 of 149.9 ± 10.6 ml in adults (n = 27, *p* < 0.05) (Fig. 1L).

### Weight and metabolic panel studies in adult and aged rhesus macaques

**Figure 2:**
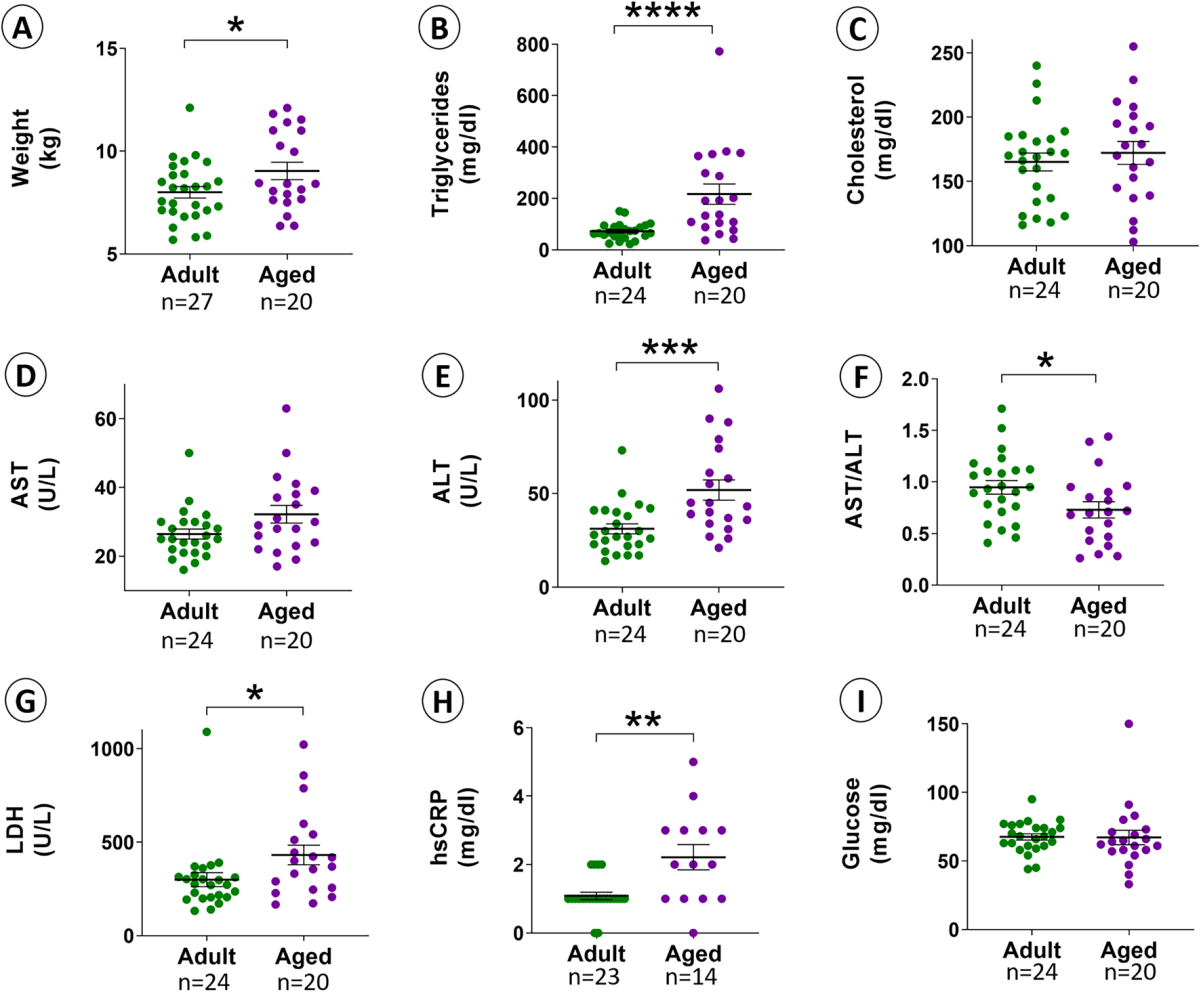
Weight and metabolic panel studies in adult and aged rhesus macaques. Body weight was significantly higher in the aged compared to adult rhesus macaques (*p* < 0.05) (**A**). There was a statistical elevation of triglycerides (*p* < 0.0001), ALT (*p* < 0.001), LDH (*p* < 0.05), and hsCRP (*p* < 0.01) in the aged cohort compared to adult animals (**B**, **E**, **G**, **H**), whereas the AST/ALT ratio was reduced in aged animals compared to the adults (*p* < 0.05) (**F**). There was no detectable difference between the groups for cholesterol, AST, and glucose levels (**C**, **D**, **I**). Data are presented as mean ± SE, and statistics are shown as * indicating *p* < 0.05, ** indicating *p* < 0.01, *** indicating *p* < 0.001, and **** indicating *p* < 0.0001 between groups.

Body weight and an expanded metabolic panel were obtained from the same cohorts of adult and aged rhesus macaques that underwent urodynamic studies. The aged subjects showed a significantly higher body weight of 9.0 ± 0.4 kg (n = 20) compared to the corresponding weight of 8.0 ± 0.3 kg (n = 27, *p* < 0.05) in adults (Fig. 2A). The BCS was 2.98 ± 0.10 (n = 27) for the adult animals and not statistically different from a BCS of 3.27 ± 0.15 (n = 20) for the aged group (*p* = 0.08). Comprehensive laboratory studies showed a significantly elevated triglyceride level of 216.8 mg/dl in aged subjects (n = 20) compared to the corresponding level of 72.1 in adults (n = 24, *p* < 0.0001) (Fig. 2B), whereas the levels for cholesterol in adult and aged subjects were not significantly different at 165.1 ± 6.9 mg/dl (n = 24) and 172.2 ± 8.9 mg/dl (n = 20), respectively (Fig. 2C). Aspartate aminotransferase (AST) was not statistically different between adult and aged subjects at 26.5 ± 1.4 U/L (n = 24) and 32.2 ± 2.5 U/L (n = 20), respectively (Fig. 2D), whereas alanine aminotransferase (ALT) was significantly elevated at 51.8 ± 5.4 U/L in aged subjects (n = 20) compared to the corresponding level of 31.2 ± 2.7 U/L in adults (n = 24) (Fig. 2E). The AST/ALT ratio was significantly reduced in aged subjects at 0.73 ± 0.08 (n = 20) compared to the corresponding ratio of 0.95 ± 0.07 in adults (n = 24) (Fig. 2F). Lactate dehydrogenase (LDH) was significantly elevated at 431.2 ± 52.3 U/L in aged subjects (n = 20) compared to the corresponding level of 299.3 ± 37.4 U/L in adults (n = 24, *p* < 0.05) (Fig. 2G). High-sensitivity C-reactive protein (hsCRP) was significantly higher at 2.2 ± 0.4 mg/dl in the aged subjects (n = 14) compared to the corresponding value of 1.1 ± 0.1 mg/dl in adults (n = 23, *p* < 0.01) (Fig. 2H). Glucose levels were not significantly different between the groups at 67.5 ± 2.3 mg/dl in adults (n = 24) and 67.2 ± 5.3 mg/dl in aged animals (n = 20) (Fig. 2I). In addition, the mean values for sodium, creatinine, blood urea nitrogen (BUN), alkaline phosphatase, gamma-glutamyl transferase, calcium, albumin, and fibrinogen were within the range of normal and not different between the adult and aged groups, whereas potassium was significantly higher at 4.01 ± 0.18 in the aged group (n = 20) compared to the corresponding value of 3.67 ± 0.06 (n = 24) in adults (*p* < 0.05).

### Urodynamic features of a cohort of aged outliers

**Figure 3:**
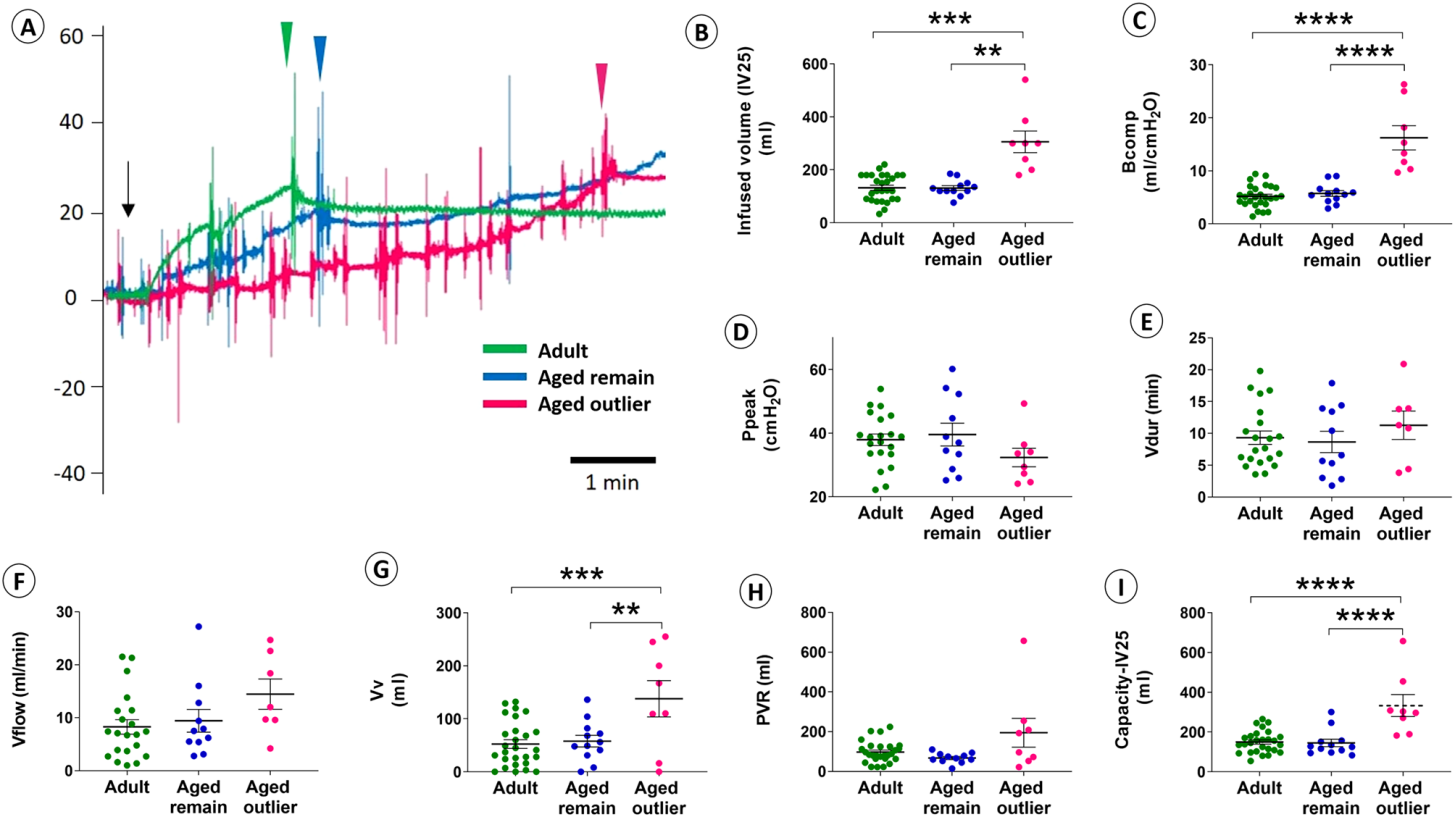
Urodynamic studies in adult, aged remain, and aged outlier rhesus macaques. Reflex micturition was evoked and shown by filling cystometry and pressure flow studies (**A**). The black arrow indicates start of bladder infusion, and the green, blue, and red arrow heads indicate end of infusion to reach target P∆ fill of 25 cm H_2_O in representative adult, aged remain, and aged outlier animals. Infused volumes were 120 ml in the adult, 120 ml in the aged remain, and 300 ml in the aged outlier subjects. Transient pressure spikes reflect movement artifacts during bladder infusion. There was a significant increase of IV25 for aged outliers compared to the aged remain group (*p* < 0.01) and adults (*p* < 0.001) but no difference between the adult and aged remain groups (**B**). There was a significant increase of Bcomp for aged outliers compared to the aged remain group (*p* < 0.0001) and adults (*p* < 0.0001) but no difference between the adult and aged remain groups (**C**). There was no detectable difference for Ppeak, Vdur, or Vflow between any of the cohorts (**D**, **E**, **F**). There was a significant increase in Vv for aged outliers compared to aged remain (*p* < 0.01) and adults (*p* < 0.001) but no difference between the adult and aged remain groups (**G**). There was no detectable difference for PVR between any of the cohorts (**H**). There was significant elevation of Capacity-IV25 for aged outliers compared to the aged remain (*p* < 0.0001) and adult (*p* < 0.0001) groups but no difference between the adult and aged remain groups (**I**). Data plots are presented as mean ± SE, and statistics are shown as * indicating *p* < 0.05, ** indicating *p* < 0.01, *** indicating *p* < 0.001, and **** indicating *p* < 0.0001 between groups.

The initial urodynamic studies identified a sub-group of outliers within the cohort of aged rhesus macaques. The outliers were defined by a Bcomp exceeding the upper range of values for the adult group in the present study. Cystometry and pressure flow studies from the aged outliers (n = 8) were compared to the remaining subjects in the aged cohort (aged remain) (n = 12) and adults (n = 27) (Fig. 3). The presence of outliers was not explained by site differences, as outliers were encountered in both the CNPRC and WNPRC cohorts of aged animals, and there was no statistical difference in the outlier proportions between the sites.

#### Filling cystometry

IV25 was significantly larger at 305.6 ± 40.7 ml in the aged outliers (n = 8) compared to the corresponding volume of 131.3 ± 8.8 ml in remaining aged subjects (n = 12, p < 0.01) and IV25 of 132.5 ± 9.7 ml in adults (n = 27, *p* < 0.001) (Fig. 3B). Bcomp was significantly higher at 16.2 ± 2.3 ml/cmH_2_O in aged outliers (n = 8) compared to corresponding Bcomp of 5.7 ± 0.5 ml/cmH_2_O in remaining aged subjects (n = 12; *p* < 0.0001) and 5.2 ± 0.4 ml/cmH_2_O in adults (n = 27, *p* < 0.001), respectively (Fig. 3C).

#### Pressure flow studies

Ppeak was not statistically different at 32.4 ± 2.9 cmH_2_O, 39.6 ± 3.6 cmH_2_O, and 37.9 ± 1.8 cmH_2_O for aged outliers (n = 8), remaining aged subjects (n = 11), and adults (n = 21), respectively (Fig. 3D). Vdur was 9,3 ± 1.0 min, 8.7 ± 1.7 min, and 11.3 ± 2.2 min for the adult (n = 21), aged remain (n = 11), and aged outlier (n = 7) cohorts, respectively, and not statistically different between groups (Fig. 3E). Vflow was 8.3 ± 1.4 ml/min, 9.4 ± 2.1 ml/min, and 14.5 ± 2.9 ml/min for the adult (n = 21), aged remain (n = 11), and aged outlier (n = 7) cohorts, respectively, and not statistically different between groups (Fig. 3F). Vv was statistically increased at 137.8 ± 34.2 ml for aged outliers (n = 8) compared to Vv of 57.9 ± 11.1 ml for remaining aged subjects (n = 12, *p* < 0.01) and 52.3 ± 8.3 ml for adults (n = 27, *p* < 0.001) (Fig. 3G). PVR 97.6 ± 10.3 ml, 67.6 ± 7.8 ml, and 194.8 ± 72.2 ml for the adult (n = 27), aged remain (n = 11), and aged outlier (n = 8) cohorts, respectively, and not statistically different (Fig. 3H). Capacity-IV25 was statistically larger at 332.5 ± 55.1 ml in aged outliers (n = 8) compared to the corresponding Capacity-IV25 for remaining aged subjects at 144.9 ± 18.7 ml (n = 12, *p* < 0.0001) and adults at 149.9 ± 10.6 ml (n = 27, *p* < 0.0001) (Fig. 3I).

### Principal component analysis

Principal component analysis (PCA) was performed to identify possible clusters and outliers among the adult and aged rhesus macaques. Thirteen variables associated with DU or metabolic syndrome were selected for each subject and included age, weight, IV25, Bcomp, Capacity-IV25, glucose, ALT, AST, AST/ALT ratio, LDH, cholesterol, triglycerides, and hsCRP. The PC analysis allowed for each animal to be represented by a single data point in a 2-dimensional space, expressed as the first and second principal components (PC1 and PC2).

**Figure 4:**
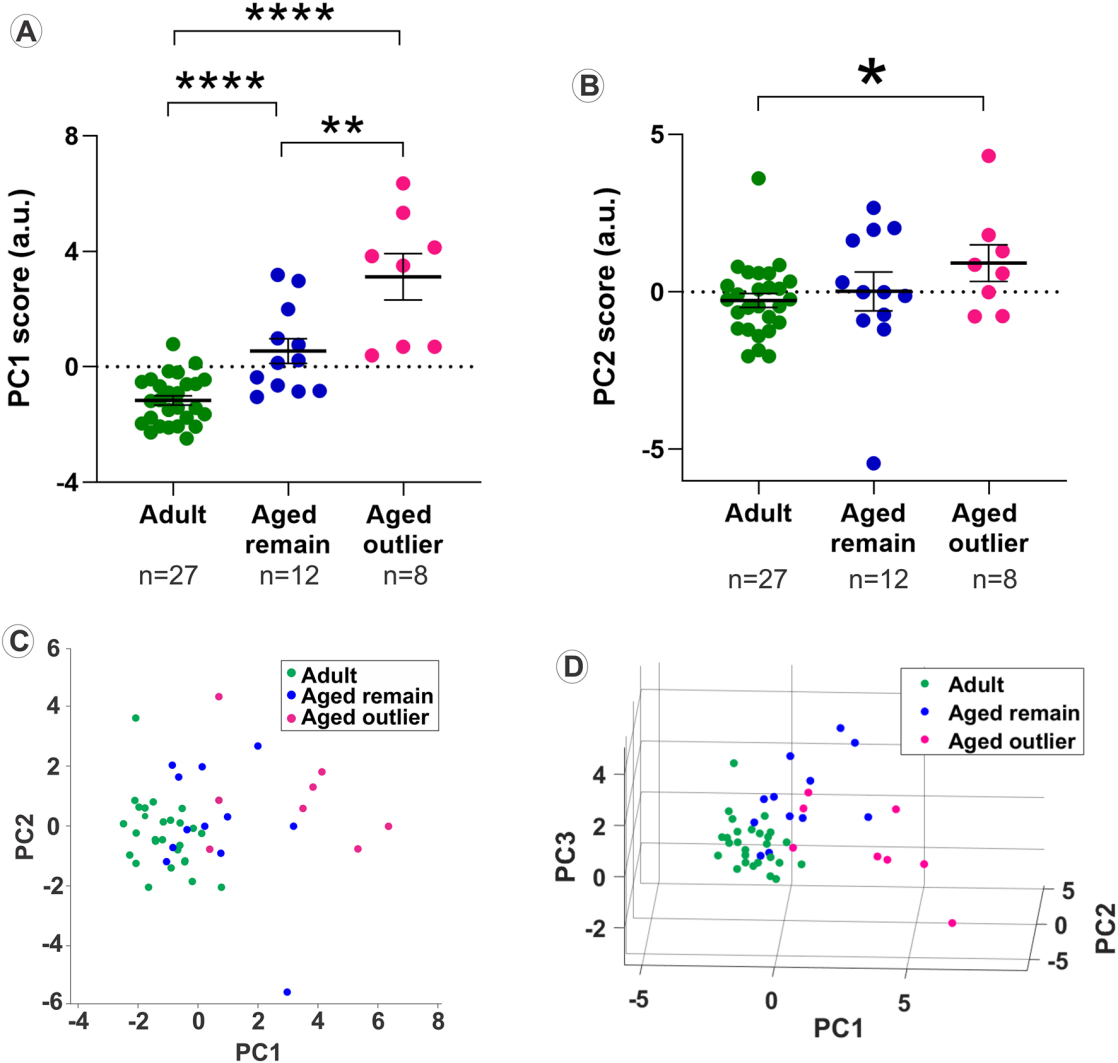
Principal component analysis. PC1 scores were significantly higher in aged outliers compared to both aged remain (*p* < 0.01) and adult (*p* < 0.0001) groups, and the PC1 score was also higher in aged remain compared to adults (*p* < 0.0001) (**A**). PC2 scores were significantly higher between aged outliers and adults (*p* < 0.05), and no differences in PC2 scores were detected between the aged remain group and adults or aged outliers (**B**). 2D-distrubtion plot of PC1 and PC2 scores showed separation of experimental groups with the majority of adult and aged remain animals forming a cluster and the majority of aged outliers forming a separate cluster (**C**). The addition of PC3 scores to provide a 3D-distribution plot to suggest separation of adults and aged outliers also in the third dimension (**D**). Data plots are presented as mean ± SE, and statistics are shown as * indicating *p* < 0.05, ** indicating *p* < 0.01, and **** indicating *p* < 0.0001 between groups.

The mean PC1 score for the aged outlier group was 3.11 ± 0.80 (n = 8) and significantly higher than the corresponding PC1 score of 0.54 ± 0.42 (n = 12) for the aged remain group (*p* < 0.01) and − 1.16 ± 0.16 (n = 27) for adults (0.0001) (Fig. 4A). The mean PC1 score was also significantly higher for the aged remain group compared to adults (*p* < 0.0001) (Fig. 4A). The mean PC2 score for the Aged outlier group was 0.91 ± 0.58 (n = 8) and significantly higher than the corresponding PC2 score of − 0.27 + 0.22 (n = 27) for adults (*p* < 0.05) but not statistically different from the mean PC2 score of 0.01 + 0.61 (n = 12) in the Aged remain group (Fig. 4B). The mean PC2 score was not statistically different between the Aged remain and Adult groups (Fig. 4B).

Cartesian representation of principal component (PC) 1 and 2 for each subject allowed for a display of the data set in two dimensions (2D) (Fig. 4C). Each subject was assigned into one of three groups. The adult subjects formed one group, and the aged subjects were divided into two separate groups based on urodynamic features. Specifically, aged subjects with a Bcomp beyond the largest Bcomp for adults were separated out as aged outliers, whereas the remaining aged subjects (aged remain) with a Bcomp within the corresponding range for adults formed a third group. Interestingly, the vast majority of adult and aged remain animals were separated into the same 2D cluster when considering PC1 and PC2, whereas the majority of aged outliers populated a different 2D cluster (Fig. 4C). When considering the 3D representation of PC1, PC2, and PC3, it is noted that the adult subjects and aged remains are separated in the third dimension (PC3) (Fig. 4D). The variability of PC1, PC2, and PC3 is 32.8%, 18.8%, and 14.8%, respectively. Therefore, the 2D display accounts for over 50% of the dataset variability, and the 3D representation includes over 66% of total dataset variability.

### Demographics

**Figure 5:**
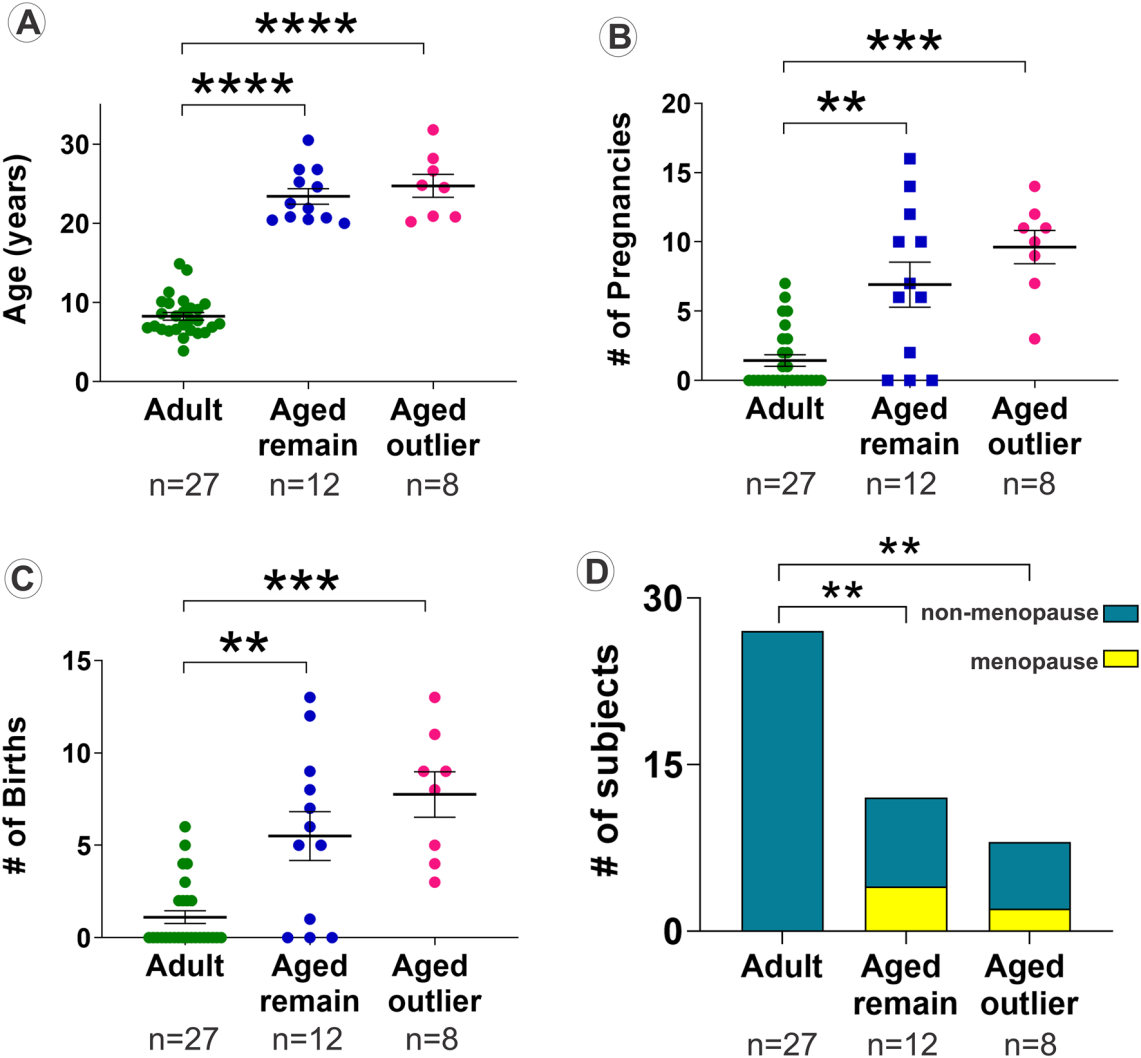
Demographics. By experimental design, both aged remain and aged outlier cohorts were significantly older compared to the adult animals (*p* < 0.0001), but there was no detectable difference in age between the aged remain and aged outlier groups (**A**). The number of prior pregnancies was significantly higher for aged remain (*p* < 0.01) and aged outliers (*p* < 0.001) compared to adult animals, but there was no detectable difference in the number of pregnancies between the aged remain and aged outlier groups (**B**). The number of births was also significantly higher for aged remain (*p* < 0.01) and aged outliers (*p* < 0.001) compared to adult animals, but there was no detectable difference in the number of births between the aged remain and aged outlier groups (**C**). A significant proportion of animals demonstrated menopause in the aged remain (*p* < 0.01) and aged outlier groups (*p* < 0.01) compared to adult animals, but there was no difference in the proportions of post-menopausal animals between the aged remain and aged outlier groups (**D**). Data plots are presented as mean ± SE, and statistics are shown as ** indicating *p* < 0.01, *** indicating *p* < 0.001, and **** indicating *p* < 0.0001 between groups.

Adults, aged remain, and aged outliers were 8.2 ± 0.5 (n = 27), 23.4 ± 1.0 (n = 12), and 24.7 ± 1.4 (n = 8) years old, respectively. Although the aged subjects were significantly older than the adults by study design (*p* < 0.0001), there was no difference in age between the aged remain and aged outliers (Fig. 5A). The number of prior pregnancies was 1.4 ± 0.4, 6.9 ± 1.6, and 9.6 ± 1.2 for adults (n = 27), aged remain (n = 12), and aged outliers (n = 8), respectively, with the number of prior pregnancies significantly higher in aged remain (*p* < 0.01) and aged outliers (*p* < 0.001) compared to adults (Fig. 5B). There was no difference in the number of prior pregnancies between aged remain and aged outliers. The number of live births was 1.1 ± 0.3, 5.5 ± 1.3, and 7.8 ± 1.2 for adults (n = 27), aged remain (n = 12), and aged outliers (n = 8), respectively, with the number of live births significantly higher in aged remain (*p* < 0.01) and aged outliers (*p* < 0.001) compared to adults (Fig. 5C). There was no difference in the number of live births between aged remain and aged outliers. All adult animals (n = 27) showed an active estrous cycle as demonstrated by periodic menstruations, whereas 2 of 8 aged remain and 4 of 12 aged outliers had entered menopause (Fig. 5D). The proportions of post-menopausal animals in the aged outlier and aged remain groups were both statistically increased compared to adults (*p* < 0.01), whereas there was no difference in the proportions of post-menopausal subjects between the two aged cohorts (Fig. 5D). Weights were 8.0 ± 0.3 (n = 27), 8.8 ± 0.6 (n = 12), and 9.3 ± 0.7 (n = 8) for the adult, aged remain, and aged outlier cohorts, respectively, and not statistically different between groups. In addition, BCS was 2.98 ± 0.10 (n = 27), 3.30 ± 0.18 (n = 12), and 3.25 ± 0.25 (n = 8) for the adult, aged remain, and aged outlier cohorts, respectively, and not statistically different between groups.

### Correlation matrix for demographic, urodynamic, and metabolic outcomes

**Figure 6:**
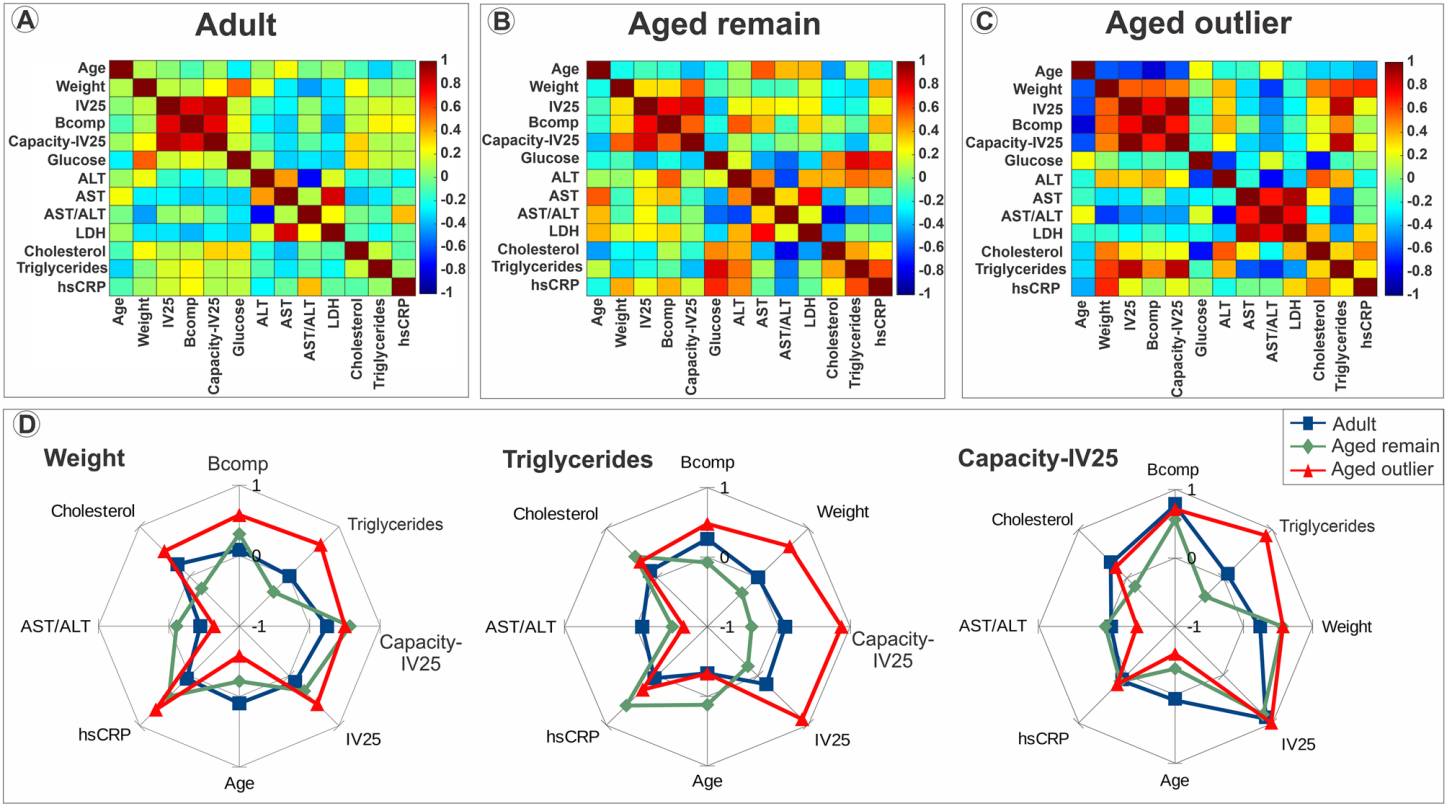
Correlation matrix for demographic, urodynamic, and metabolic outcomes. Heat map display shows correlation pairs across multiple demographic, urodynamic, and metabolic outcomes, including age, weight, IV25, Bcomp, Capacity-IV25, glucose, ALT, AST, AST/ALT ratio, LDH, cholesterol, triglycerides, and hsCRP (**A**–**C**). Note a higher number of pairs with strong correlations for the aged outliers compared to adult and aged remain groups (**C**). Radar plot displays to show correlations between weight, triglycerides, and Capacity-IV25 with multiple outcome measures, including also age, Bcomp, IV25, cholesterol, AST/ALT, and hsCRP (**D**). A positive or negative correlation over 0.5 is considered a strong correlation. Weight, triglycerides, and Capacity-IV25 showed mostly modest correlations with the other markers for the adult and aged remain groups, whereas weight, triglycerides, and Capacity-IV25 showed multiple strong correlations with other markers of detrusor underactivity and metabolic syndrome, including IV25, Bcomp, AST/ALT ratio, and hsCRP (**D**).

Pairwise correlations were next performed between multiple demographic, urodynamic and laboratory markers, including age, weight, IV25, Bcomp, Capacity-IV25, glucose, ALT, AST AST/ALT, LDH, Cholesterol, Triglycerides, and hsCRP for the Adult, Adult remain, and Aged outlier groups (Fig. 6). A heat map display suggested an increased number of pairs with a strong correlation between individual outcome measures for the aged outliers (Fig. 6C) compared to the adult and aged remain groups (Fig. 6A,B). The heat map display included several unique pairs of data indicating strong correlations between weight, bladder capacity and compliance, and metabolic markers for the aged outlier group (Fig. 6C). Next, we determined how weight, triglyceride, and Capacity-IV25 were correlated to each other and to IV25, Bcomp, cholesterol, AST/ALT, and hsCRP. A correlation of 0.50 or higher in either direction was considered to indicate a strong association between pairs. A strong correlation was detected between weight and Capacity-IV25 in both Aged remain and Aged outlier groups, whereas a strong correlation between weight and Bcomp, IV25, cholesterol, triglycerides, and AST/ALT ratio was only detected in the Aged outlier group (Fig. 6D; Supplementary Table 1). Collectively, the correlation matrix studies showed a strong correlation between urodynamic indicators for detrusor underactivity, including increased bladder capacity and compliance, and demographic and metabolic markers, such as increased weight, hypertriglyceridemia, decreased AST/ALT ratio, and increased hsCRP for the aged outlier group.

## Discussion

Spontaneous DU was demonstrated by urodynamic studies in a cohort of aged rhesus macaques. The detrusor impairment was associated with weight and laboratory markers for metabolic syndrome in the aged subjects. However, urodynamic function in aged primates without a detectable metabolic disturbance was indistinguishable from corresponding LUT assessments in healthy adult subjects. The findings were unaffected by the estrous cycle, as parity and menopause status were not different between the aged cohorts with and without DU. Collectively, these results suggest metabolic syndrome as a novel and previously unknown contributor to DU in the elderly. The findings may guide future clinical studies on the potential causal relationship between metabolic syndrome, a risk factor that can be subject to mitigation, and the development of an underactive bladder (UAB).

Approximately 20 million men and women in the United States are affected by UAB, and a very large portion of this underserved patient group is found within the elderly population^5,29^. The clinical symptoms of UAB include an increased bladder capacity, poor sensation of bladder fullness, reduced contractility of the detrusor, and impaired emptying of the bladder^29–32^. Almost half of older men and women that undergo a comprehensive medical evaluation for LUT symptoms show signs of DU^33,34^. The health implications from a diagnosis of DU are significant, as UAB markedly increases the risk for urinary tract infections and hospitalizations as well as reduces quality of life and community independence, especially in the elderly population.

The diagnosis of DU is based on urodynamic studies and, when demonstrated, generally supports the diagnosis of the clinical symptom complex of UAB^35,36^. It is recognized that UAB represents a clinical condition in humans and that animal models are principally based on studies of DU, using cystometrogram (CMG) recordings for functional assessments. However, uniform diagnostic criteria for DU based on CMG studies are also not well established but subject to ongoing evaluations and discussions^2,37^. To date, most experimental studies on DU have been performed in rats, mice and guinea pigs to determine the effects of disease or neurological trauma, including studies on the effects of diabetes, nerve root and peripheral nerve injuries, and bladder outlet obstruction^38–41^. Aging studies on LUT function in rodents are valuable but limited, in part due to a much shorter life span compared to humans. In contrast, the present study provides a new large animal model for spontaneous development of DU with aging in rhesus macaques, as demonstrated by CMG findings of increased bladder capacity and compliance in the aged animals.

In the present studies, the aged rhesus macaques showed increased body weight compared to adult animals, suggestive of obesity. The weight measurements did not take into account body frame size or abdominal circumference. However, animal fitness and muscularity were considered in BCS assessments^24–26^ but did not show statistical significance (*p* < 0.08) between the adult and aged cohorts. In future studies of metabolic effects on autonomic functions, rhesus macaques will also undergo calculation of body mass index (BMI) based on weight and crown-to-rump length (CRL)^42^ as quantitative indicators of fitness and obesity. An additional limitation in the present study is the absence of blood pressure comparisons between groups, as hypertension is an indicator of metabolic syndrome. Blood pressure measurements at times of experimental procedures will be included as added outcome measures in future studies.

Although urodynamic outcome measures, terminology, and diagnostic criteria for DU, including consideration of both passive and active properties of the detrusor^43^, were used for the present translational research study in NHPs, there are important distinctions between human and animal studies on DU to consider. For instance, in human studies, CMG studies are performed with an awake subject and with subject interactions, including voluntary activation of voiding upon instructions. In our rhesus macaque studies, the animals are under a light plane of sedation provided by ketamine, and the active phase of the study is performed using partial bladder filling to evoke the activation of micturition reflexes and voiding. The influence of anesthesia is known to suppress micturition reflexes and maximum peak pressure as a result of voluntary emptying of the bladder, including the use of, for instance, accessory abdominal muscles is not possible in most current animal models. Comparisons of LUT properties and function between the aged and adult cohorts of rhesus macaques are still possible, as standard operating procedures for the evaluation of passive detrusor properties and evoked micturition reflexes were used in the present study^27^.

Non-human primates are the phylogenetically closest relatives of humans and provide physiologically important and clinically relevant models for the evaluation of pathobiology and therapeutics in metabolic disease research^44^. For instance, rhesus macaques may develop obesity, metabolic syndrome, and diabetes mellitus with aging^19,45^. Dysmetabolic and aging rhesus macaques also develop spontaneous liver steatosis and non-alcoholic fatty liver disease (NAFLD), validated by ultrasound and liver biopsy studies^46,47^. Additional laboratory markers for NAFLD includes elevated triglycerides and ALT with a reduced AST/ALT ratio^48^. Elevated levels of hsCRP, a marker of inflammation, is primarily produced in the liver, and is also detected in the setting of metabolic syndrome and NAFLD^49,50^.

In captivity-maintained male and female cynomolgus monkeys (*Macaca fascicularis*), body weight increases rapidly during the first 7–8 years of life, stabilizes over the next few years, and shows a gradual decrease in aged animals^51,52^. In the present study, in contrast, the aged primates demonstrated an increased body weight compared to adult animals, suggestive of an age-associated obesity. A similar aged-associated obesity has been seen in prior aging studies in rhesus macaques in captivity^53^. The aged cohort also showed elevated triglycerides, ALT, LDH, and hsCRP, as well as a decreased AST/ALT ratio, suggestive of NAFLD. Collectively, the present findings provide clinical and laboratory support for the development of metabolic syndrome in the aged rhesus macaques. By experimental design, no animals with diabetes mellitus were included in the studies, and the findings support to the notion that elevation of glucose levels represents a later sign in the development of metabolic syndrome^54^. In addition, electrolyte studies showed an isolated elevation of potassium in the aged rhesus macaque group but in the absence of any changes in creatinine and BUN to suggest any concurrent renal disease. However, both hyper- and hypokalemia have associated with aging^55–57^ and a possible role for potassium in the pathobiology associated with metabolic syndrome remains uncertain^58^. In future studies, a glucose tolerance test will be included to validate the absence of diabetes mellitus in animals of the experimental series, and the metabolic panel will be expanded to also include leptin and insulin levels as these biomarkers have been associated with LUT and detrusor dysregulation in the setting of metabolic syndrome^59,60^.

One limitation of the present study is the inclusion of only female and no male animals. LUT function is known to be highly sexually dimorphic in both humans and non-human primates^27,61^, and metabolic syndrome is prevalent in both men and women across all sociodemographic groups in the United States^62^. It is therefore possible that sex may represent a biological variable for a possible association between metabolic syndrome and DU. Future studies on, for instance, metabolic syndrome risk factor interventions or treatments of DU in animal models or clinical studies will benefit from the inclusion of both men and women.

A significant finding of the present studies is that aging alone may not be sufficient for the development of DU or UAB. The aged rhesus macaques separated into two cohorts, with and without signs of CMG features of DU, including increased bladder capacity and compliance. Interestingly, the aged primates with CMG-supported signs of DU showed a strong association by PCA and paired correlations with weight and blood chemistry markers for metabolic syndrome, whereas the aged animals with a normal urodynamic evaluation showed unremarkable weight and metabolic chemistry outcomes, which were not different from corresponding findings in healthy adult rhesus macaques. Earlier clinical studies have suggested an association between metabolic syndrome and overactive bladder (OAB) syndrome, characterized clinically by urinary urgency, frequency, and nocturia, as well as by detrusor overactivity by CMG recordings^63–65^. However, the present identification of metabolic syndrome as a putative risk factor for DU and UAB is novel and has translational research interest and implications, as metabolic syndrome may be amenable to risk factor modification and prevention in clinical practice.

## Supplementary Information

Supplementary Table 1.

## Data Availability

Any raw data supporting the current study is available from the corresponding author upon reasonable request.

## Code availability

The code used in the calculations, the input and output data, information regarding their development, and directions for code use are available at https://github.com/petrabartmeyer/PCA_analysis.
