# Supplementary material for: Detrusor underactivity is associated with metabolic syndrome in aged primates
Source: Sci Rep. 2023 Apr 25;13:6716. doi: 10.1038/s41598-023-33112-3 (PMC10130177; doi:10.1038/s41598-023-33112-3)
Supplement: Supplementary file 1 — Supplementary Table 1. [file 41598_2023_33112_MOESM1_ESM.docx]

**Supplementary Table 1. Correlation pairs across multiple demographic, urodynamic, and metabolic outcomes.** Outcome measures for adult, aged remain, and aged outlier groups of rhesus macaques include age, weight, IV25, Bcomp, Capacity-IV25, glucose, ALT, AST, AST/ALT ratio, LDH, cholesterol, triglycerides, and hsCRP. A positive or negative correlation over 0.5 is considered a strong correlation. Weight, triglycerides, and Capacity-IV25 are biological markers associated with metabolic syndrome and DU.

|  |  | Weight | Triglycerides | Cap-IV25 | IV25 | Age | hsCRP | AST/ALT | Cholesterol | Bcomp |
| --- | --- | --- | --- | --- | --- | --- | --- | --- | --- | --- |
| Weight | Adult |  | 0.01 | 0.25 | 0.12 | 0.1 | 0.05 | -0.45 | 0.24 | 0.08 |
|  | Aged remain |  | -0.31 | 0.57 | 0.3 | -0.22 | 0.41 | -0.11 | -0.24 | 0.31 |
|  | Aged outlier |  | 0.63 | 0.5 | 0.57 | -0.58 | 0.68 | -0.64 | 0.5 | 0.58 |
| Triglycerides | Adult | 0.01 |  | 0.09 | 0.17 | -0.33 | 0.05 | -0.09 | 0.13 | 0.26 |
|  | Aged remain | -0.31 |  | -0.38 | -0.2 | 0.12 | 0.6 | -0.51 | 0.43 | -0.08 |
|  | Aged outlier | 0.63 |  | 0.88 | 0.88 | -0.33 | 0.28 | -0.67 | 0.32 | 0.48 |
| CapIV25 | Adult | 0.25 | 0.09 |  | 0.88 | 0.06 | 0.09 | -0.07 | 0.33 | 0.79 |
|  | Aged remain | 0.57 | -0.38 |  | 0.82 | -0.38 | 0.13 | 0.02 | -0.17 | 0.56 |
|  | Aged outlier | 0.58 | 0.88 |  | 0.99 | -0.6 | 0.2 | -0.44 | 0.23 | 0.71 |
